# Crystal structure and Hirshfeld surface analysis of 3-amino-5-phenyl­thia­zolidin-2-iminium bromide

**DOI:** 10.1107/S2056989019013069

**Published:** 2019-09-27

**Authors:** Gulnara Sh. Duruskari, Ali N. Khalilov, Mehmet Akkurt, Gunay Z. Mammadova, Taras Chyrka, Abel M. Maharramov

**Affiliations:** a Organic Chemistry Department, Baku State University, Z. Xalilov str. 23, Az, 1148 Baku, Azerbaijan; b Department of Physics and Chemistry, "Composite Materials" Scientific Research Center, Azerbaijan State Economic University (UNEC), H. Aliyev str. 135, Az 1063, Baku, Azerbaijan; c Department of Physics, Faculty of Sciences, Erciyes University, 38039 Kayseri, Turkey; d Department of Theoretical and Industrial Heat Engineering (TPT), National Technical University of Ukraine, "Igor Sikorsky Kyiv Polytechnic Institute", 03056, Kyiv, Ukraine

**Keywords:** crystal structure, charge-assisted hydrogen bonding, thia­zolidine ring, Hirshfeld surface analysis

## Abstract

In the cation of the title salt, C_9_H_12_N_3_S^+^·Br^−^, the thia­zolidine ring adopts an envelope conformation with the C atom adjacent to the phenyl ring as the flap. In the crystal, N—H⋯Br hydrogen bonds link the components into a three-dimensional network. Weak π–π stacking inter­actions between the phenyl rings of adjacent cations also contribute to the mol­ecular packing. A Hirshfeld surface analysis was conducted to qu­antify the contributions of the different inter­molecular inter­actions and contacts.

## Abstract 2

In the crystal of the title salt, the cations and anions are linked *via* N—H⋯Br hydrogen bonds to form a three-dimensional network.

## Chemical context   

As well as their synthetic utility, thia­zolidine derivatives possess a broad spectrum of biological activities such as anti­malarial, anti­bacterial, anti­microbial, anti-inflammatory, anti­cancer, *etc*. The biological activities of compounds containing a thia­zolidine core, such as 1,3-thia­zolidines, 2,4-dione-, 4-oxo-thia­zolidine, *etc*. were summarized in a recent review (Makwana & Malani, 2017 ▸). On the other hand, as hydrazones these N-containing ligands have been widely used in the synthesis of coordination compounds (Gurbanov *et al.*, 2018*a* ▸,*b* ▸). The non-covalent donor or acceptor properties of N-containing ligands can also contribute to their catalytic activity, among other properties (Mahmudov *et al.*, 2019 ▸; Zubkov *et al.*, 2018 ▸). As part of our ongoing work in this area, we now describe the synthesis and structure of the title mol­ecular salt, C_9_H_12_N_3_S^+^·Br^−^, (I).
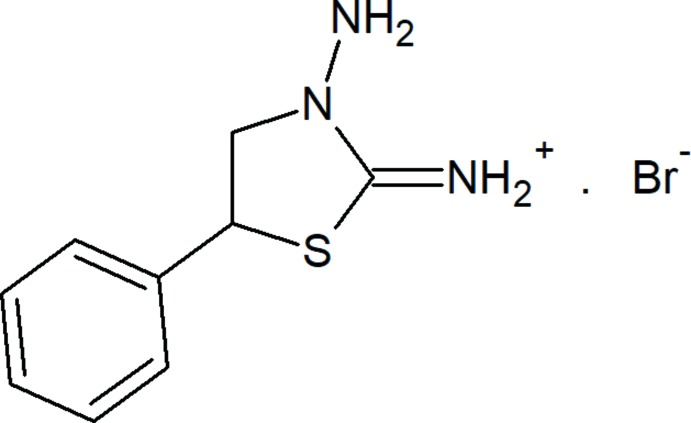


## Structural commentary   

**Figure 1:**
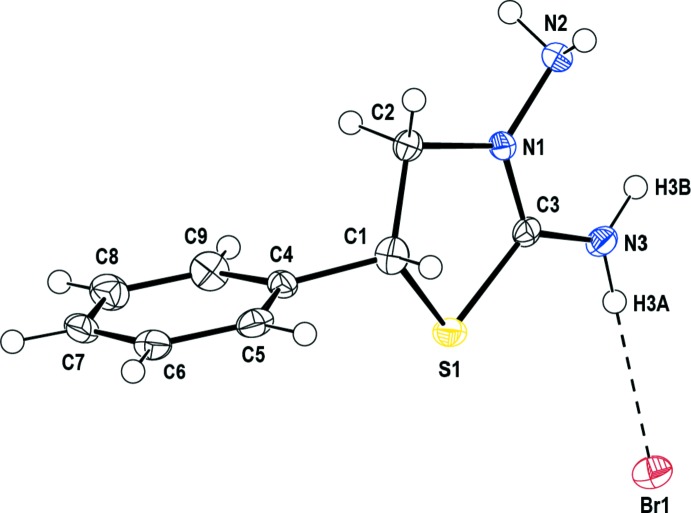
The mol­ecular structure of the title salt. Displacement ellipsoids are drawn at the 50% probability level and the H⋯Br hydrogen bond is indicated by a dashed line.

In the cation of (I) (Fig. 1 ▸), the thia­zolidine ring (S1/N1/C1–C3) adopts an envelope conformation with puckering parameters of *Q*(2) = 0.317 (2) Å and φ(2) = 225.2 (4)°: the flap atom is C1. In the arbitrarily chosen asymmetric unit, C1 has an *R* configuration, but symmetry generates a racemic mixture in the crystal. The dihedral angle between the mean plane of the thia­zolidine ring (all atoms) and the phenyl ring (C4–C9) is 89.27 (13)°.

## Supra­molecular features and Hirshfeld surface analysis   

**Figure 2:**
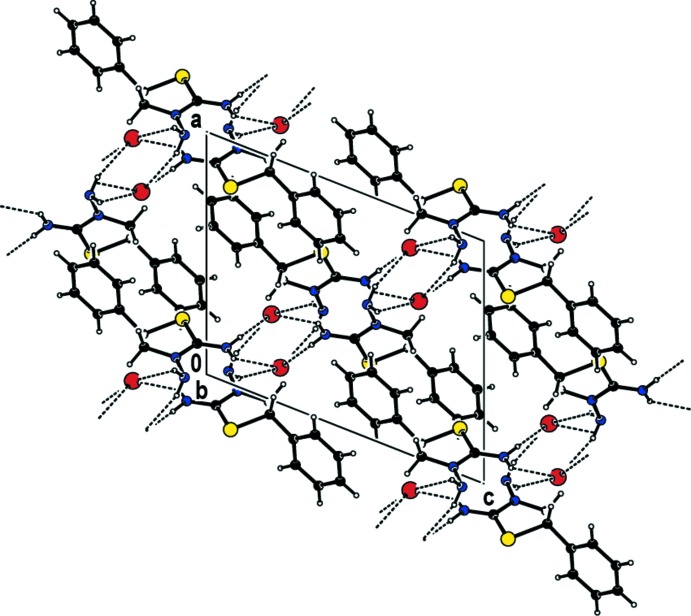
Part of the crystal structure of the title compound, showing the formation of N—H⋯Br hydrogen bonds in the *ac* plane.

In the crystal, each cation forms N—H⋯Br hydrogen bonds (Table 1 ▸) as well as aromatic π–π stacking inter­actions between the phenyl rings of adjacent cations [*Cg*2⋯*Cg*2^iv^ = 3.7758 (16) Å; symmetry code: (iv) 1 − *x*, 1 − *y*, 2 − *z*; where *Cg*2 is the centroid of the phenyl ring of the cation]: chains of cations form along the [101] direction (Fig. 2 ▸). Taking into account the hydrogen bonding and π-π stacking, the overall connectivity is three-dimensional.

**Figure 3:**
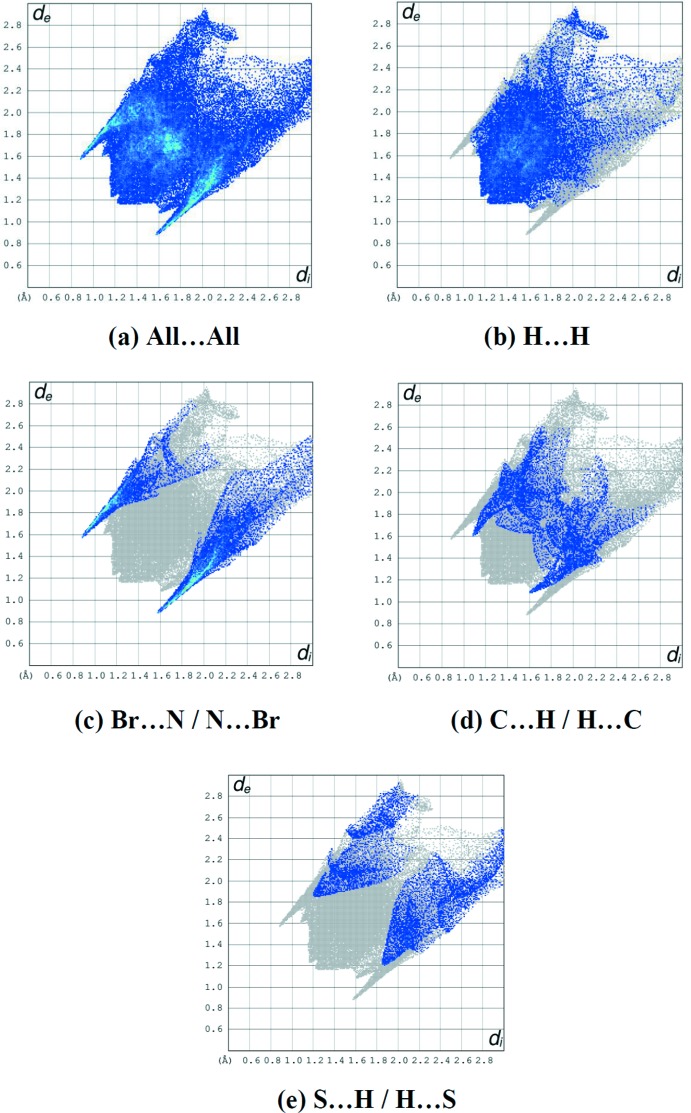
The two-dimensional fingerprint plots of the title salt, showing (*a*) all inter­actions, and delineated into (*b*) H⋯H, (*c*) Br⋯N/N⋯Br, (*d*) C⋯H/H⋯C and (*e*) S⋯H/H⋯S inter­actions [*d*_e_ and *d*_i_ represent the distances from a point on the Hirshfeld surface to the nearest atoms outside (external) and inside (inter­nal) the surface, respectively].

Hirshfeld surface analysis (Spackman & Jayatilaka, 2009 ▸; Spackman & McKinnon, 2002 ▸) was carried out with *CrystalExplorer3.1* (Wolff *et al.*, 2012 ▸) to further investigate the presence of hydrogen bonds and inter­molecular inter­actions in the crystal structure (see supporting information). Fig. 3 ▸(*a*) shows the two-dimensional fingerprint of the sum of the contacts contributing to the Hirshfeld surface represented in normal mode while those delineated into H⋯H (41.5%), Br⋯N/N⋯Br (24.1%), C⋯H/H⋯C (13.8%) and S⋯H/H⋯S (11.7%) contacts, respectively, are shown in Fig. 3 ▸ *b*–*e*. All contacts are listed in Table 2 ▸.

## Database survey   

A search of the Cambridge Structural Database (CSD, Version 5.40, February 2019; Groom *et al.*, 2016 ▸) for 2-thia­zolidiniminium compounds gave eight hits, *viz.* BOBWIB (Khalilov *et al.*, 2019 ▸), UDELUN (Akkurt *et al.*, 2018 ▸), WILBIC (Marthi *et al.*, 1994 ▸), WILBOI (Marthi *et al.*, 1994 ▸), WILBOI01 (Marthi *et al.*, 1994 ▸), YITCEJ (Martem’yanova *et al.*, 1993*a* ▸), YITCAF (Martem’yanova *et al.*, 1993*b* ▸) and YOPLUK (Marthi *et al.*, 1995 ▸).

In the crystal of BOBWIB (Khalilov *et al.*, 2019 ▸), the thia­zolidine ring adopts an envelope conformation. In the crystal, centrosymmetrically related cations and anions are linked into dimeric units *via* N—H⋯Br hydrogen bonds, which are further connected by weak C—H⋯Br hydrogen bonds into chains parallel to [110]. In the crystal of UDELUN (Akkurt *et al.*, 2018 ▸), C—H⋯Br and N—H⋯Br hydrogen bonds link the components into a three-dimensional network with the cations and anions stacked along the *b*-axis direction. Weak C—H⋯π inter­actions, which only involve the minor disorder component of the ring, also contribute to the mol­ecular packing. In addition, there are also inversion-related Cl⋯Cl halogen bonds and C—Cl⋯π(ring) contacts. In the other structures, the 3-N atom carries a C substituent: the first three crystal structures were determined for racemic (WILBIC; Marthi *et al.*, 1994 ▸) and two optically active samples (WILBOI and WILBOI01; Marthi *et al.*, 1994 ▸) of 3-(2′-chloro-2′-phenyl­eth­yl)-2-thia­zolidiniminium *p*-toluene­sulfonate. In all three structures, the most disordered fragment of these mol­ecules is the asymmetric C atom and the Cl atom attached to it. The disorder of the cation in the racemate corresponds to the presence of both enanti­omers at each site in the ratio 0.821 (3): 0.179 (3). The system of hydrogen bonds connecting two cations and two anions into 12-membered rings is identical in the racemic and in the optically active crystals. YITCEJ (Martem’yanova *et al.*, 1993*a* ▸) is a product of the inter­action of 2-amino-5-methyl­thia­zoline with methyl iodide, with alkyl­ation at the endocylic nitro­gen atom, while YITCAF (Martem’yanova *et al.*, 1993*b* ▸) is a product of the reaction of 3-nitro-5-meth­oxy-, 3-nitro-5-chloro-, and 3-bromo-5-nitro­salicyl­aldehyde with the heterocyclic base to form the salt-like complexes.

## Synthesis and crystallization   

To a solution of 2.2 mmol (0.6 g) (1,2-di­bromo­eth­yl)benzene in 20 ml of ethanol were added 2.3 mmol (0.3 g) of thio­semicarbazide hydro­chloride; 3-4 drops of piperidine were added and the mixture was refluxed for 7 h. The reaction mixture was cooled to room temperature and the solid product was precipitated from solution, collected by filtration and recrystallized from ethanol solution to give colourless crystals of (I) with a yield of 88%, m.p. = 468 K. Analysis calculated for C_9_H_12_BrN_3_S: C 39.43; H 4.41; N 15.33. Found: C 39.40; H 4.39; N 15.30%. ^1^H NMR (300 MHz, DMSO-*d*_6_) : 4.16 (*q*, 1H, CH_2_,^3^ *J*_H–H_ = 5.4); 4.45 (*t*, 1H, CH_2_, ^3^ *J*_H–H_ = 8.4); 5.25 (*t*, 1H, CH-Ar, ^3^ *J*_H–H_ = 5.4); 7.32–7.50 (*m*, 5H, 5Ar-H); 9.12 (*s*, 2H, NH_2_); 9,78 (*s*, 1H, NH=). ^13^C NMR (75 MHz, DMSO-*d*_6_ **):** 44.42, 62.06, 127.59, 128.76, 129.17, 138.85, 168.53. MS (ESI), *m*/*z*: 194.28 [C_9_H_12_N_3_S]^+^ and 79.88 Br^−^.

## Refinement   

Crystal data, data collection and structure refinement details are summarized in Table 3 ▸. All H atoms on C atoms were placed at calculated positions (C—H = 0.95–1.00 Å) and refined using a riding model. The N-bound hydrogen atoms were located from difference-Fourier maps and relocated to idealized locations (N—H = 0.90 Å) and refined as riding atoms. The constraint *U*_iso_(H) = 1.2*U*_eq_(carrier) was applied in all cases. One outlier (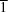
01) was omitted in the final cycles of refinement.

## Supplementary Material

Crystal structure: contains datablock(s) I. DOI: 10.1107/S2056989019013069/hb7855sup1.cif

Structure factors: contains datablock(s) I. DOI: 10.1107/S2056989019013069/hb7855Isup2.hkl

Hirshfeld surface analysis figures. DOI: 10.1107/S2056989019013069/hb7855sup3.docx

Supporting information file. DOI: 10.1107/S2056989019013069/hb7855Isup4.cml

CCDC reference: 1955268

Additional supporting information:  crystallographic information; 3D view; checkCIF report

## Figures and Tables

**Table 1:** Hydrogen-bond geometry (Å, °)

*D*—H⋯*A*	*D*—H	H⋯*A*	*D*⋯*A*	*D*—H⋯*A*
N2—H2*A*⋯Br1^i^	0.90	2.68	3.530 (2)	158
N2—H2*B*⋯Br1^ii^	0.90	2.73	3.524 (2)	148
N3—H3*A*⋯Br1	0.90	2.38	3.271 (2)	169
N3—H3*B*⋯Br1^iii^	0.90	2.56	3.337 (2)	145

Symmetry codes: (i) 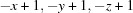; (ii) 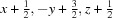; (iii) 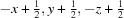.

**Table 2:** Percentage contributions of inter­atomic contacts to the Hirshfeld surface for the title salt

Contact	Percentage contribution
H⋯H	41.5
Br⋯N/N⋯Br	24.1
C⋯H/H⋯C	13.8
S⋯H/H⋯S	11.7
N⋯H/H⋯N	3.6
C⋯C	3.3
N⋯C/C⋯N	1.5
N⋯N	0.3
S⋯C/C⋯S	0.3

**Table 3:** Experimental details

Crystal data	
Chemical formula	C_9_H_12_N_3_S^+^·Br^−^
*M*_r_	274.19
Crystal system, space group	Monoclinic, *P*2_1_/*n*
Temperature (K)	150
*a*, *b*, *c* (Å)	10.5986 (5), 8.7168 (3), 13.0308 (5)
β (°)	111.513 (2)
*V* (Å^3^)	1119.99 (8)
*Z*	4
Radiation type	Mo *K*α
μ (mm^−1^)	3.82
Crystal size (mm)	0.18 × 0.14 × 0.11

Data collection	
Diffractometer	Bruker APEXII CCD
Absorption correction	Multi-scan (*SADABS*; Bruker, 2003 ▸)
*T*_min_, *T*_max_	0.534, 0.661
No. of measured, independent and observed [*I* > 2σ(*I*)] reflections	8461, 2303, 1998
*R*_int_	0.029
(sin θ/λ)_max_ (Å^−1^)	0.626

Refinement	
*R*[*F* ^2^ > 2σ(*F* ^2^)], *wR*(*F* ^2^), *S*	0.027, 0.070, 1.02
No. of reflections	2303
No. of parameters	127
H-atom treatment	H-atom parameters constrained
Δρ_max_, Δρ_min_ (e Å^−3^)	0.61, −0.33

Computer programs: *APEX2* and *SAINT* (Bruker, 2003 ▸), *SHELXT2014* (Sheldrick, 2015*a* ▸), *SHELXL2016* (Sheldrick, 2015*b* ▸), *ORTEP-3 for Windows* (Farrugia, 2012 ▸) and *PLATON* (Spek, 2003 ▸).

## supplementary crystallographic information

### Crystal data

**Table:**

C_9_H_12_N_3_S^+^·Br^−^	*F*(000) = 552
*M**_r_* = 274.19	*D*_x_ = 1.626 Mg m^−^^3^
Monoclinic, *P*2_1_/*n*	Mo *K*α radiation, λ = 0.71073 Å
*a* = 10.5986 (5) Å	Cell parameters from 3357 reflections
*b* = 8.7168 (3) Å	θ = 2.9–26.3°
*c* = 13.0308 (5) Å	µ = 3.82 mm^−^^1^
β = 111.513 (2)°	*T* = 150 K
*V* = 1119.99 (8) Å^3^	Block, colorless
*Z* = 4	0.18 × 0.14 × 0.11 mm

### Data collection

**Table:**

Bruker APEXII CCD diffractometer	1998 reflections with *I* > 2σ(*I*)
φ and ω scans	*R*_int_ = 0.029
Absorption correction: multi-scan (SADABS; Bruker, 2003)	θ_max_ = 26.4°, θ_min_ = 2.9°
*T*_min_ = 0.534, *T*_max_ = 0.661	*h* = −13→13
8461 measured reflections	*k* = −10→10
2303 independent reflections	*l* = −16→16

### Refinement

**Table:**

Refinement on *F*^2^	Primary atom site location: structure-invariant direct methods
Least-squares matrix: full	Secondary atom site location: difference Fourier map
*R*[*F*^2^ > 2σ(*F*^2^)] = 0.027	Hydrogen site location: mixed
*wR*(*F*^2^) = 0.070	H-atom parameters constrained
*S* = 1.02	*w* = 1/[σ^2^(*F*_o_^2^) + (0.0381*P*)^2^ + 0.5896*P*] where *P* = (*F*_o_^2^ + 2*F*_c_^2^)/3
2303 reflections	(Δ/σ)_max_ = 0.001
127 parameters	Δρ_max_ = 0.61 e Å^−^^3^
0 restraints	Δρ_min_ = −0.33 e Å^−^^3^

### Special details

**Table:**

Geometry. All esds (except the esd in the dihedral angle between two l.s. planes) are estimated using the full covariance matrix. The cell esds are taken into account individually in the estimation of esds in distances, angles and torsion angles; correlations between esds in cell parameters are only used when they are defined by crystal symmetry. An approximate (isotropic) treatment of cell esds is used for estimating esds involving l.s. planes.

### Fractional atomic coordinates and isotropic or equivalent isotropic displacement parameters (Å^2^)

**Table:**

	*x*	*y*	*z*	*U*_iso_*/*U*_eq_	
Br1	0.14662 (3)	0.38142 (3)	0.26547 (2)	0.02369 (10)	
S1	0.31116 (7)	0.46985 (7)	0.58677 (5)	0.02034 (15)	
N1	0.4802 (2)	0.6893 (2)	0.61070 (15)	0.0159 (4)	
N2	0.5487 (2)	0.8083 (2)	0.57955 (15)	0.0183 (4)	
H2A	0.635551	0.776693	0.607350	0.022*	
H2B	0.535901	0.891753	0.615230	0.022*	
N3	0.3577 (2)	0.6293 (2)	0.42850 (16)	0.0179 (4)	
H3A	0.292719	0.572157	0.379225	0.021*	
H3B	0.390509	0.709317	0.402415	0.021*	
C1	0.4372 (3)	0.5007 (3)	0.72776 (19)	0.0214 (5)	
H1A	0.513995	0.427032	0.741377	0.026*	
C2	0.4890 (3)	0.6645 (3)	0.72463 (19)	0.0189 (5)	
H2C	0.432008	0.739798	0.744749	0.023*	
H2D	0.583832	0.675080	0.776759	0.023*	
C3	0.3879 (2)	0.6085 (3)	0.53385 (19)	0.0154 (5)	
C4	0.3746 (2)	0.4760 (3)	0.81375 (18)	0.0168 (5)	
C5	0.4280 (3)	0.3584 (3)	0.8912 (2)	0.0212 (5)	
H5A	0.497585	0.293462	0.886358	0.025*	
C6	0.3773 (3)	0.3379 (3)	0.97571 (19)	0.0208 (5)	
H6A	0.412191	0.258553	1.028582	0.025*	
C7	0.2769 (3)	0.4330 (3)	0.9816 (2)	0.0225 (5)	
H7A	0.244208	0.420521	1.039905	0.027*	
C8	0.2230 (3)	0.5463 (3)	0.9041 (2)	0.0265 (6)	
H8A	0.152610	0.610477	0.908407	0.032*	
C9	0.2715 (3)	0.5662 (3)	0.8203 (2)	0.0249 (6)	
H9A	0.233208	0.643424	0.766388	0.030*	

### Atomic displacement parameters (Å^2^)

**Table:**

	*U*^11^	*U*^22^	*U*^33^	*U*^12^	*U*^13^	*U*^23^
Br1	0.02460 (15)	0.02084 (15)	0.02181 (15)	−0.00181 (11)	0.00401 (11)	−0.00181 (10)
S1	0.0263 (3)	0.0175 (3)	0.0173 (3)	−0.0062 (3)	0.0081 (2)	−0.0012 (2)
N1	0.0197 (10)	0.0165 (10)	0.0132 (9)	−0.0036 (8)	0.0078 (8)	−0.0004 (8)
N2	0.0208 (11)	0.0162 (10)	0.0198 (10)	−0.0030 (9)	0.0099 (9)	−0.0004 (8)
N3	0.0239 (11)	0.0151 (10)	0.0144 (10)	−0.0011 (9)	0.0066 (8)	−0.0014 (8)
C1	0.0215 (13)	0.0233 (13)	0.0184 (12)	0.0033 (11)	0.0061 (10)	0.0023 (10)
C2	0.0225 (13)	0.0215 (12)	0.0126 (11)	−0.0041 (10)	0.0064 (10)	−0.0018 (10)
C3	0.0176 (12)	0.0121 (11)	0.0178 (12)	0.0027 (9)	0.0082 (10)	−0.0009 (9)
C4	0.0171 (12)	0.0193 (12)	0.0131 (11)	−0.0054 (10)	0.0046 (9)	−0.0010 (9)
C5	0.0175 (12)	0.0183 (12)	0.0255 (13)	−0.0020 (10)	0.0051 (10)	−0.0066 (10)
C6	0.0244 (13)	0.0193 (12)	0.0147 (12)	−0.0050 (10)	0.0027 (10)	0.0016 (10)
C7	0.0215 (13)	0.0253 (13)	0.0214 (13)	−0.0100 (11)	0.0085 (11)	−0.0029 (11)
C8	0.0208 (13)	0.0258 (14)	0.0335 (15)	−0.0012 (11)	0.0108 (12)	−0.0007 (12)
C9	0.0219 (13)	0.0246 (13)	0.0266 (14)	0.0041 (11)	0.0070 (11)	0.0036 (11)

### Geometric parameters (Å, º)

**Table:**

S1—C3	1.735 (2)	C2—H2C	0.9900
S1—C1	1.853 (2)	C2—H2D	0.9900
N1—C3	1.318 (3)	C4—C9	1.374 (4)
N1—N2	1.408 (3)	C4—C5	1.404 (3)
N1—C2	1.469 (3)	C5—C6	1.403 (4)
N2—H2A	0.9000	C5—H5A	0.9500
N2—H2B	0.9000	C6—C7	1.373 (4)
N3—C3	1.303 (3)	C6—H6A	0.9500
N3—H3A	0.9000	C7—C8	1.378 (4)
N3—H3B	0.9000	C7—H7A	0.9500
C1—C4	1.513 (3)	C8—C9	1.378 (4)
C1—C2	1.535 (4)	C8—H8A	0.9500
C1—H1A	1.0000	C9—H9A	0.9500
			
C3—S1—C1	91.16 (11)	N3—C3—N1	123.6 (2)
C3—N1—N2	119.48 (18)	N3—C3—S1	123.08 (18)
C3—N1—C2	116.3 (2)	N1—C3—S1	113.33 (17)
N2—N1—C2	123.48 (18)	C9—C4—C5	119.6 (2)
N1—N2—H2A	102.6	C9—C4—C1	122.7 (2)
N1—N2—H2B	104.8	C5—C4—C1	117.7 (2)
H2A—N2—H2B	111.4	C6—C5—C4	119.2 (2)
C3—N3—H3A	120.2	C6—C5—H5A	120.4
C3—N3—H3B	121.7	C4—C5—H5A	120.4
H3A—N3—H3B	117.4	C7—C6—C5	119.6 (2)
C4—C1—C2	114.2 (2)	C7—C6—H6A	120.2
C4—C1—S1	111.16 (17)	C5—C6—H6A	120.2
C2—C1—S1	104.09 (16)	C6—C7—C8	120.9 (2)
C4—C1—H1A	109.1	C6—C7—H7A	119.5
C2—C1—H1A	109.1	C8—C7—H7A	119.5
S1—C1—H1A	109.1	C9—C8—C7	119.7 (3)
N1—C2—C1	105.85 (19)	C9—C8—H8A	120.1
N1—C2—H2C	110.6	C7—C8—H8A	120.1
C1—C2—H2C	110.6	C4—C9—C8	120.9 (2)
N1—C2—H2D	110.6	C4—C9—H9A	119.5
C1—C2—H2D	110.6	C8—C9—H9A	119.5
H2C—C2—H2D	108.7		
			
C3—S1—C1—C4	147.17 (19)	C2—C1—C4—C9	53.9 (3)
C3—S1—C1—C2	23.78 (17)	S1—C1—C4—C9	−63.5 (3)
C3—N1—C2—C1	26.7 (3)	C2—C1—C4—C5	−124.2 (2)
N2—N1—C2—C1	−163.3 (2)	S1—C1—C4—C5	118.4 (2)
C4—C1—C2—N1	−152.1 (2)	C9—C4—C5—C6	−1.7 (4)
S1—C1—C2—N1	−30.7 (2)	C1—C4—C5—C6	176.5 (2)
N2—N1—C3—N3	1.9 (3)	C4—C5—C6—C7	−0.2 (4)
C2—N1—C3—N3	172.3 (2)	C5—C6—C7—C8	1.5 (4)
N2—N1—C3—S1	−178.62 (16)	C6—C7—C8—C9	−0.9 (4)
C2—N1—C3—S1	−8.2 (3)	C5—C4—C9—C8	2.2 (4)
C1—S1—C3—N3	169.2 (2)	C1—C4—C9—C8	−175.8 (2)
C1—S1—C3—N1	−10.29 (19)	C7—C8—C9—C4	−1.0 (4)

### Hydrogen-bond geometry (Å, º)

**Table:**

*D*—H···*A*	*D*—H	H···*A*	*D*···*A*	*D*—H···*A*
N2—H2*A*···Br1^i^	0.90	2.68	3.530 (2)	158
N2—H2*B*···Br1^ii^	0.90	2.73	3.524 (2)	148
N3—H3*A*···Br1	0.90	2.38	3.271 (2)	169
N3—H3*B*···Br1^iii^	0.90	2.56	3.337 (2)	145

Symmetry codes: (i) −*x*+1, −*y*+1, −*z*+1; (ii) *x*+1/2, −*y*+3/2, *z*+1/2; (iii) −*x*+1/2, *y*+1/2, −*z*+1/2.
