# Supplementary material for: Crystal structure and Hirshfeld surface analysis of 3-amino-5-phenyl­thia­zolidin-2-iminium bromide
Source: Acta Crystallogr E Crystallogr Commun. 2019 Sep 27;75(Pt 10):1544–7. doi: 10.1107/S2056989019013069 (PMC6775745; doi:10.1107/S2056989019013069)
Supplement: Supplementary file 3 [file e-75-01544-sup3.docx]

| **hb7855-supplement** |
| --- |

| **Crystal structure and Hirshfeld surface analysis of 3-amino-5-phenylthiazolidin-2-iminium bromide** |
| --- |

| **Gulnara Sh. Duruskari,^a^ Ali N. Khalilov,^b^ Mehmet Akkurt,^c^ Gunay Z. Mammadova,^a^ Taras Chyrka^d^* and Abel M. Maharramov^a^**  **^a^**Organic Chemistry Department, Baku State University, Z. Xalilov str. 23, Az, 1148 Baku, Azerbaijan, **^b^**Organic Chemistry Department, Baku State University, Z. Xalilov str. 23, Az, 1148 Baku, Azerbaijan, and, Department of Physics and Chemistry, "Composite Materials" Scientific, Research Center, Azerbaijan State Economic University (UNEC), H. Aliyev str. 135, Az 1063, Baku, Azerbaijan, **^c^**Department of Physics, Faculty of Sciences, Erciyes University, 38039 Kayseri, Turkey, and **^d^**Department of Theoretical and Industrial Heat Engineering (TPT), National Technical University of Ukraine "Igor Sikorsky Kyiv Polytechnic, Institute", 03056, Kyiv, Ukraine |
| --- |

| [Correspondence e-mail: **mustford@ukr.net**](SIMON%20_publ_contact_author_email) |
| --- |

|  |
| --- |

| 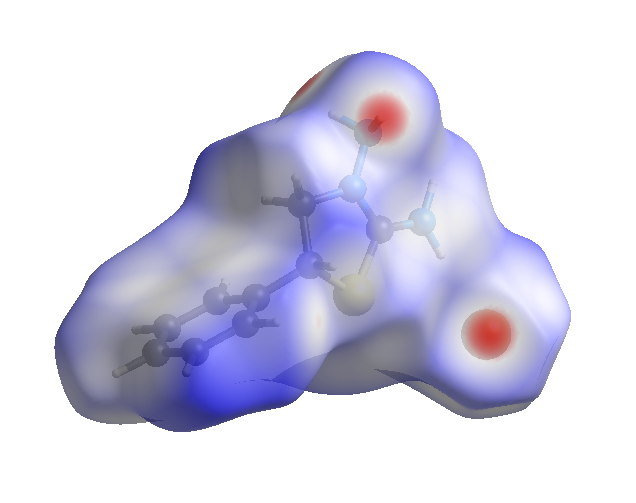  [**Figure S1**](readonly) Hirshfeld surface of the title salt mapped with *d_norm_*.  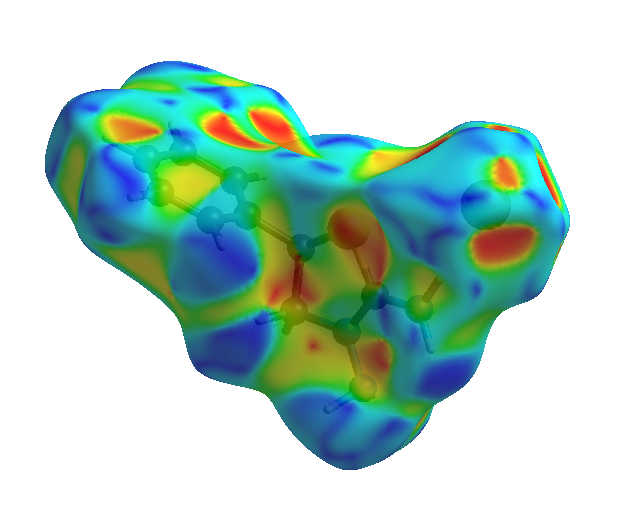  [**Figure S2**](readonly) Hirshfeld surface of the title salt mapped with shape index.    [**Figure S3**](readonly) The Hirshfeld surfaces mapped over d_norm_ in the title salt, showing (*a*) all interactions, and delineated into (*b*) H···H, (*c*) Br···N/N···Br, (*d*) C···H/H···C and (*e*) S···H/H···S interactions [*d_e_* and *d_i_* represent the distances from a point on the Hirshfeld surface to the nearest atoms outside (external) and inside (internal) the surface, respectively]. |
| --- |
